# Adult stress exposure blunts dopamine system hyperresponsivity in a neurodevelopmental rodent model of schizophrenia

**DOI:** 10.1038/s41537-022-00235-x

**Published:** 2022-03-25

**Authors:** Millie Rincón-Cortés, Anthony A. Grace

**Affiliations:** 1 Departments of Neuroscience, University of Pittsburgh, Pittsburgh, PA 15260 USA; 2 Departments of Psychiatry, University of Pittsburgh, Pittsburgh, PA 15260 USA; 3 Departments of Psychology, University of Pittsburgh, Pittsburgh, PA 15260 USA; 4 Present Address: Department of Neuroscience, School of Behavioral Brain Sciences, University of Texas at Dallas, Richardson, TX USA

**Keywords:** Schizophrenia, Psychiatric disorders

## Abstract

Stress is a major risk factor for the development of both schizophrenia and depression, and comorbidity between the two is common in schizoaffective disorders. However, the effects of stress exposure (i.e. chronic mild stress-CMS) on depression-related phenotypes in a neurodevelopmental model relevant to schizophrenia (i.e. methylazoxymethanol acetate—MAM) have yet to be explored and could provide insight into shared mechanisms of disease. To this end, we combined the prenatal MAM model with adult CMS exposure and explored the resultant pathophysiology using the social approach test (SAT), immobility in the forced swim test (FST) and amphetamine-induced hyperlocomotion (AIH) as depression- and schizophrenia-related endophenotypes and performed extracellular recordings of ventral tegmental area (VTA) DA neurons. MAM rats exhibited a reduction in social approach and increased VTA DA neuron activity compared to SAL rats or CMS groups. Separate cohorts of MAM animals were subjected to FST and AIH testing (counterbalanced order) or FST only. CMS groups exhibited increased FST immobility. Post-FST, both MAM groups (MAM-CON, MAM-CMS) exhibited blunted locomotor response to amphetamine compared with their SAL counterparts exposed to the same tests. Post-FST, MAM rats exhibited comparable VTA population activity to SAL rats, and CMS groups exhibited attenuated VTA population activity. Apomorphine administration results were consistent with the model suggesting that reductions in VTA DA neuron activity in MAM rats following FST exposure resulted from over-excitation, or depolarization block. These data suggest stress-induced DA downregulation in MAM rats, as FST exposure was sufficient to block the DA hyperresponsivity phenotype.

## Introduction

Depression and schizophrenia can be comorbid disorders, suggesting a relationship between them that affects the risk and severity for both^1^. Indeed, schizophrenia patients have a higher risk for developing depression and greater functional impairment^1–4^, and depressed patients exhibit a higher risk of developing psychosis^5^. Furthermore, negative symptoms of schizophrenia and depressive symptoms often overlap. For example, anhedonia and social avoidance are often reported in both depression and schizophrenia^6–9^. The mutual relationship of risk and the common psychopathology-related behavioural phenotypes between both disorders suggest overlap in the aetiology of the depression and schizophrenia^10^. Chronic stress exposure is a major risk factor for the development of both schizophrenia and depression^11–16^ and effects may depend on the timing of the insult^17,18^. However, little is known regarding the effects of adult chronic stress exposure in animal models relevant for the study of schizophrenia, which could provide insights into mechanisms underlying comorbidity between depression and schizophrenia.

Depression and schizophrenia are characterized by dysregulation of dopamine (DA) system function^18–21^. Depression is associated with mesolimbic DA system hyporesponsivity^20–22^. For example, rats exposed to chronic mild stress (CMS) or learned helplessness (LH), two widely used rodent models of stress-induced adaptations useful for the study of depression, exhibit reduced numbers of spontaneously active DA neurons (i.e. population activity) in the medial ventral tegmental area (VTA), which projects to reward-related regions of the ventral striatum^23^, and greater immobility duration in the forced swim test (FST)^22,24–26^. In contrast, schizophrenia is linked to associative DA system hyper-responsivity^27,28^. Rats treated prenatally with the mitotoxin methylazoxymethanol acetate (MAM) on gestational day (GD) 17, a neurodevelopmental model relevant to schizophrenia, exhibit increases in adult DA neuron population activity primarily in the lateral VTA that projects to the associative striatum^29^, which are correlated with amphetamine-induced hyperlocomotion (AIH)^27,30^. Similarly, neuroimaging studies in schizophrenia patients have demonstrated greater amphetamine-induced DA release within the striatum, and that the amplitude of DA release correlates with exacerbation of psychosis^31,32^. Thus, post-pubertal altered DA responsivity to psychomotor stimulants is commonly observed in schizophrenia patients and MAM rats^33^. Taken together, these data demonstrate that alterations in VTA DA neuron activity in rodents are associated with distinct schizophrenia and depression-related behavioural phenotypes.

Importantly, both CMS and MAM are useful animal models for the study of depression and schizophrenia, respectively, because they fulfill three types of validity when modeling psychiatric disorders: face (i.e. similar symptomatology), construct (i.e. similar genetic/environmental cause) and predictive (i.e. can be used to make predictions about the human phenomenon of interest and responds to relevant drug treatments and/or therapies)^34,35^. Thus, the presence of both schizophrenia and depression-related endophenotypes in a single model may be useful in understanding comorbidity and provide insight into shared mechanisms of disease. However, the comorbid effects of prenatal MAM treatment and adult CMS exposure have not yet been explored. Therefore, we tested the effects of combined prenatal MAM administration with adult CMS exposure on social sniff time in the social approach test (SAT), FST immobility and AIH as schizophrenia (SAT, AIH) and depression-related (SAT, FST) behavioural phenotypes, and conducted electrophysiological recordings of DA neurons within the VTA.

## Results

### Effects of prenatal MAM and adult CMS on social behaviour and VTA activity

**Fig. 1:**
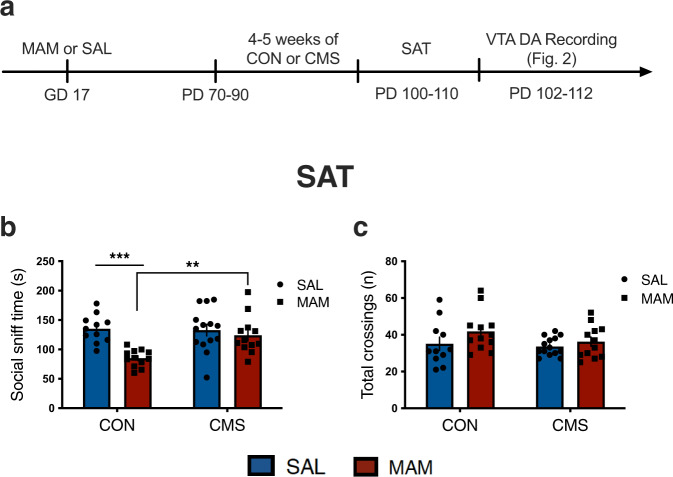
Rats treated with prenatal MAM exhibit attenuated social motivation. **a** Timeline of experimental design. **b** MAM-CON animals exhibited reduced social sniff time compared with vehicle-treated controls (SAL-CON) and MAM-CMS rats (two-way ANOVA interaction followed by Tukey’s: *p* < 0.05, *n* = 11–14 per group). **b** No effect of MAM treatment (*p* = 0.09) or CMS exposure (*p* = 0.20) was found for total number of crossings, suggesting no impact on locomotor activity. Error bars represent mean ± SEM. Blue bars and circles represent SAL animals; red bars and squares represent MAM animals. CON groups are plotted on the left and CMS groups are plotted on the right. **p < 0.01, ***p < 0.001.

Rats were tested in the SAT following 4–5 weeks of CMS exposure (SAL-CON: *n* = 11, MAM-CON: *n* = 12, SAL-CMS: *n* = 14, MAM-CMS: *n* = 12). A significant interaction between prenatal MAM treatment and adult CMS exposure was found (two-way ANOVA; F_1,45_ = 6.20; *p* < 0.05). MAM-CON rats exhibited reduced social sniff time (85.09 ± 14.55 s) compared to control rats (SAL-CON: 135 ± 23.73 s) and MAM-CMS rats (124 ± 32.58 s) (Tukey’s; *p* < 0.001 and *p* < 0.01, respectively; Fig. 1a). No effect of MAM (two-way ANOVA; F_1,45_ = 2.93; *p* = 0.09) or CMS (two-way ANOVA; F_1,45_ = 1.72; *p* = 0.20) was found for total number of chamber crossings (Fig. 1b), suggesting comparable locomotor activity between groups.

**Fig. 2:**
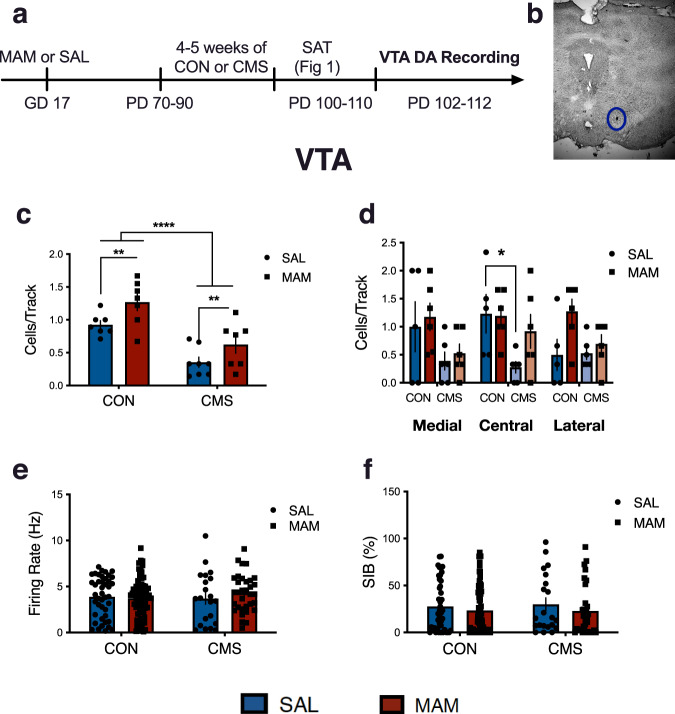
Adult CMS exposure reduces VTA population activity in both SAL and MAM rats. **a** Timeline and experimental design. **b** Representative placement of VTA DA neurons (electrode placement inside blue circle). **c** Main effects of prenatal MAM treatment (two-way ANOVA: *p* < 0.01) and adult CMS exposure (two-way ANOVA: *p* < 0.0001) were found for population activity (*n* = 7–8 per group). Both groups of MAM animals exhibited increased numbers of spontaneously active VTA DA neurons (i.e. cells/track-CPT) compared to SAL animals. CMS exposure attenuated VTA population activity, as indexed by reduced cells/track in SAL-CMS and MAM-CMS rats compared to standard-housed animals (SAL-CON, MAM-CON). **d** Population activity was reduced along the central tracks of the VTA in SAL-CMS rats compared to SAL-CON rats (*p* < 0.05). **e** No effect of prenatal MAM treatment (*p* = 0.56) or adult CMS exposure (*p* = 0.61) was found for basal firing rate. **f** No effect of prenatal MAM treatment (*p* = 0.21) or adult CMS exposure (*p* = 0.84) was found for percentage of spikes occurring in bursts (SIB%). Error bars represent mean ± SEM. Blue bars and circles represent SAL animals; red bars and squares represent MAM animals. CON groups are plotted on the left and CMS groups are plotted on the right. *p < 0.05, **p < 0.01, ****p < 0.0001.

Electrophysiological recordings were conducted 2–7 days post-testing in the same animals tested for social behaviour (SAL-CON: *n* = 7, 43 neurons; MAM-CON: *n* = 7, 40 neurons; SAL-CMS: *n* = 8, 21 neurons; MAM-CMS: *n* = 7, 34 neurons**;** Fig. 2a, b). Main effects of prenatal MAM treatment (two-way ANOVA; F_1,25_ = 8.78; *p* < 0.01) and adult CMS exposure (two-way ANOVA; F_1,25_ = 34.6; *p* < 0.0001) were found for population activity (i.e. cells/track-CPT) (Fig. 2c). Both groups of MAM animals (MAM-CON: 1.27 ± 0.34 CPT, MAM-CMS: 0.62 ± 0.35 CPT) exhibited increased numbers of spontaneously active VTA DA neurons compared to SAL animals (SAL-CON: 0.93 ± 0.16 CPT, SAL-CMS: 0.35 ± 0.22 CPT). Moreover, both CMS groups (SAL-CMS, MAM-CMS) exhibited attenuated population activity, as indexed by reduced cells/track, compared to standard-housed animals (SAL-CON, MAM-CON). Given evidence that the VTA is functionally segregated^23^, the cells/track data were analyzed according to location in the medial, central or lateral VTA (Fig. 2d). Population activity was reduced in the central VTA tracks of SAL-CMS rats compared with SAL-CON rats (F_3,19_ = 8.884; *p* < 0.05). No effect of MAM treatment (two-way ANOVA; F_1,167_ = 0.33; *p* = 0.56) or CMS (two-way ANOVA; F_1,167_ = 0.26; *p* = 0.61) was found for basal firing rate (Fig. 2e) or for percentage of spikes occurring in bursts (two-way ANOVA; MAM: F_1,167_ = 1.58; *p* = 0.21; CMS: F_1,167_ = 0.04; *p* = 0.84; Fig. 2f).

### Effects of prenatal MAM and adult stress on FST immobility and locomotor response to amphetamine

**Fig. 3:**
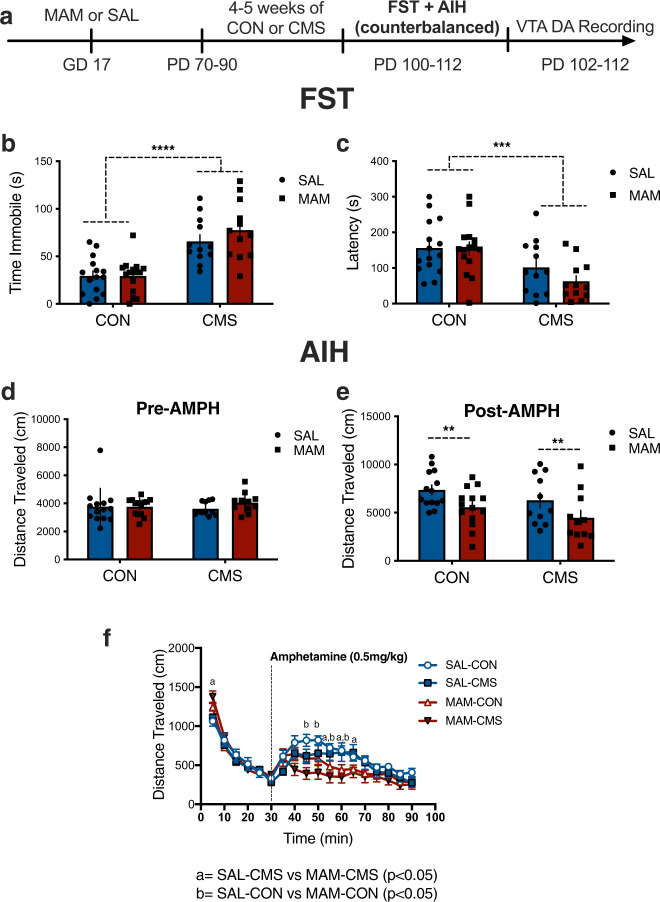
Behavioural effects of prenatal MAM treatment and adult CMS exposure on forced swim (FST) and amphetamine-induced hyperlocomotion (AIH). **a** Timeline and experimental design. SAL and MAM animals exposed to adult CMS were tested in the FST and for AIH in a counterbalanced order (~1 week in between tests) and exhibited **b** increased immobility duration in the FST (*p* < 0.0001) and **c** reduced latency to immobility (*p* < 0.001) compared to SAL-CON and MAM-CON animals (main effect of CMS: *p* < 0.05, *n* = 12–15 per group). **d** No effect of MAM treatment (*p* = 0.38) or adult CMS exposure (*p* = 0.79) was found for total distance traveled at baseline (30 min). **e** Post-FST, a main effect of MAM treatment was found on total distance traveled over 60 min following amphetamine (AMPH) injection (one-way ANOVA: *p* < 0.01, *n* = 11–14 per group). Both MAM groups (i.e. MAM-CON, MAM-CMS) exhibited blunted locomotor responses to AMPH compared to SAL groups. **f** Specifically, MAM-CON rats exhibited blunted locomotor response to AMPH compared to SAL-CON rats at 15, 20, 25 and 30 min post-AMPH injection; MAM-CMS rats exhibited blunted locomotor response to AMPH compared with SAL-CMS rats at 25, 30 and 35 min post-injection. Blue bars and circles represent SAL animals; red bars and squares represent MAM animals. CON groups are plotted on the left and CMS groups are plotted on the right. **p < 0.01, ***p < 0.001, ****p < 0.0001.

Following 4–5 weeks of CMS or control conditions, separate cohorts of SAL and MAM rats were tested for their locomotor response to amphetamine and in the FST in a counterbalanced order 1 week apart (SAL-CON: *n* = 15, SAL-CMS: *n* = 12, MAM-CON: *n* = 15, MAM-CMS: *n* = 12**;** Fig. 3a). Adult CMS exposure increased FST immobility (two-way ANOVA; F_1,50_ = 44.88; *p* < 0.001) and reduced latency to immobility (two-way ANOVA; F_1,50_ = 13.88; *p* < 0.001) (Fig. 3b, c). Thus, SAL-CMS and MAM-CMS rats exhibited greater immobility duration (Fig. 3b) and reduced latency to immobility compared with SAL-CON and MAM-CON rats (Fig. 3c).

Rats exposed to the FST underwent testing for AIH the following week. No effect of MAM (two-way ANOVA; F_1,46_ = 0.7693; *p* = 0.38) or CMS (two-way ANOVA; F_1,46_ = 0.0762; *p* = 0.79) was found for total distance traveled at baseline (i.e. prior to AMPH injection) (Fig. 3d). Post-FST, an effect of MAM was found for total distance traveled (2-way ANOVA; F_1,46_ = 8.089; *p* < 0.01) following AMPH injection. MAM-CON rats and MAM-CMS exhibited a reduction in total distance traveled compared to SAL-CON and SAL-CMS rats, respectively (Fig. 3e). Specifically, MAM-CON rats exhibited blunted locomotor response to AMPH compared to SAL-CON rats at 15, 20, 25 and 30 min post-injection; MAM-CMS rats exhibited blunted locomotor response to AMPH compared with SAL-CMS rats at 25, 30 and 35 min post-injection (Fig. 3F). Collectively, these data suggest that acute stress (FST) exposure is sufficient to blunt AIH previously reported in MAM rats^27,30,36,37^.

### Electrophysiological evidence for acute and chronic stress-induced attenuation of VTA activity in MAM rats

**Fig. 4:**
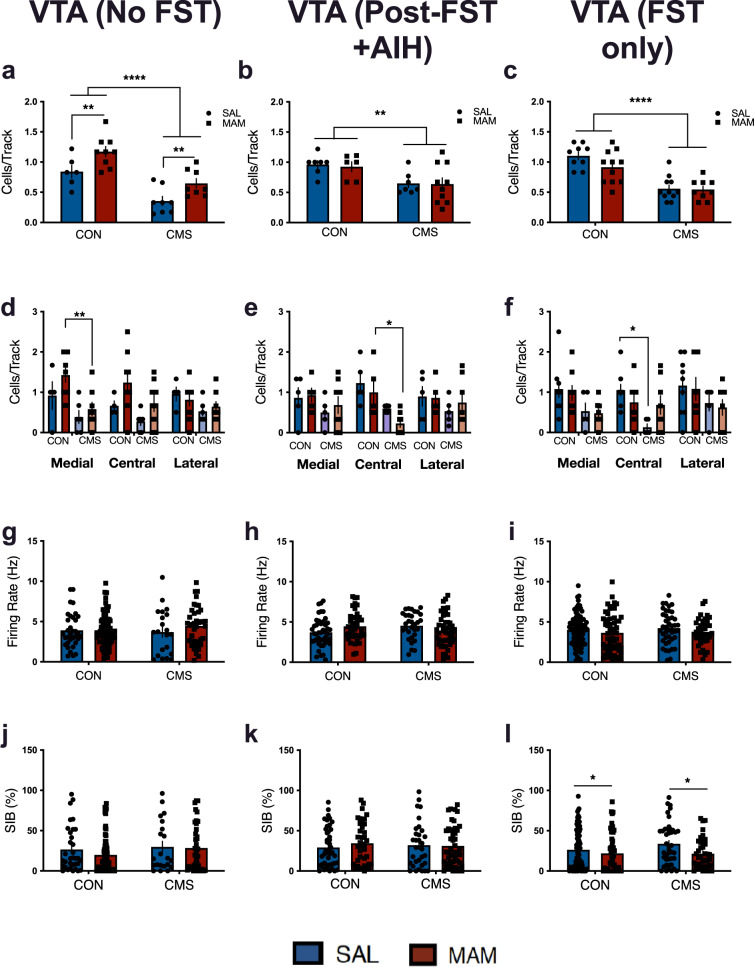
FST exposure eliminates increased VTA population activity in MAM rats compared to SAL rats. **a** At baseline, MAM-CON rats exhibited elevated numbers of DA cells/track compared to SAL-CON and both CMS groups (two-way ANOVA: main effect of MAM (*p* < 0.01) and adult CMS (*p* < 0.0001), *n* = 6–9 per group). **b** After FST and AIH exposure, there was no difference between SAL and MAM rats in both CON and CMS conditions, although both SAL-CMS and MAM-CMS rats exhibited blunted VTA population activity compared to SAL-CON and MAM-CON rats (two-way ANOVA; main effect CMS: *p* < 0.01; *n* = 7–9). **c** Post-FST only, both CMS groups (SAL-CMS, MAM-CMS) exhibited blunted VTA population activity compared to CON groups (SAL-CON, MAM-CON (two-way ANOVA; main effect CMS: *p* < 0.0001; *n* = 9–11). **d** At baseline, population activity was reduced along the medial tracks of the VTA in MAM-CMS rats compared to MAM-CON rats (*p* < 0.05). **e** Post-FST and AIH, population activity was reduced along the central tracks of the VTA in MAM-CMS rats compared to MAM-CON rats (*p* < 0.01). **f** Post-FST only, population activity was reduced along the central tracks of the VTA in SAL-CMS rats compared to SAL-CON rats (*p* < 0.05). **g** No effect of MAM (*p* = 0.31) or CMS (*p* = 0.68) was found for firing rate at baseline. **h** No effect of MAM (*p* = 0.71) or CMS (*p* = 0.43) was found for firing rate post-FST and AIH. **i** Post-FST only, there was no effect of MAM (*p* = 0.14) or CMS (*p* = 0.58) for firing rate. **j** At baseline, no effect of MAM (*p* = 0.37) or CMS (*p* = 0.19) was found for burst firing. **k** No effect of MAM (*p* = 0.62) or CMS (*p* = 0.95) was found for burst firing post-FST and AIH. **l** Post-FST only, an effect was found for MAM (*p* = 0.02) but not CMS (*p* = 0.30). Error bars represent mean ± SEM. Blue bars and circles represent SAL animals; red bars and squares represent MAM animals. CON groups are plotted on the left and CMS groups are plotted on the right. *p < 0.05, **p < 0.01, ****p < 0.0001.

Electrophysiological recordings were conducted 2–7 days following the last behavioural test (FST or AIH). This time frame was selected to be consistent with previous studies looking at CMS effects on VTA activity^22,25^. In contrast to post-SAT (Fig. 2) and baseline (i.e. no FST) conditions (main effect of MAM: F_1,27_ = 13.04; *p* < 0.01; main effect of CMS: F_1,27_ = 34.36; *p* < 0.0001; SAL-CON: *n* = 6, 34 neurons; MAM-CON: *n* = 9, 72 neurons; SAL-CMS: *n* = 7, 43 neurons; MAM-CMS: *n* = 8, 39 neurons; Fig. 4a), in which MAM rats showed higher cells/track in both CON and CMS groups (MAM-CON: 1.15 ± 0.18 CPT; MAM-CMS: 0.65 ± 0.22 CPT vs SAL-CON: 0.84 ± 0.25 CPT; SAL-CMS: 0.36 ± 0.22 CPT): Fig. 4a), after FST and AIH testing there was no difference between SAL and MAM rats in CON or CMS conditions (Fig. 4b). Post-FST and AIH, a main effect of CMS was found for VTA population activity (two-way ANOVA; F_1,26_ = 11.07; *p* < 0.01; SAL-CON: *n* = 7, 44 neurons; MAM-CON: *n* = 6, 39 neurons; SAL-CMS: *n* = 7, 32 neurons; MAM-CMS: *n* = 10, 46 neurons; Fig. 4b). Rats from both CMS groups (SAL-CMS: 0.65 ± 0.18 CPT; MAM-CMS: 0.64 ± 0.33 CPT) exhibited lower numbers of active DA neurons per electrode track compared with controls (SAL-CON: 0.96 ± 0.17 CPT; MAM-CON: 0.93 ± 0.20 CPT). As an additional control, we conducted electrophysiological recordings in a group of animals that were exposed only to the FST (no AIH) and found a main effect of CMS (two-way ANOVA; F_1,36_ = 44; *p* < 0.0001; SAL-CON: *n* = 9, 88 neurons; MAM-CON: *n* = 11, 55 neurons; SAL-CMS: *n* = 11, 42 neurons; MAM-CMS: *n* = 9, 37 neurons; Fig. 4c). Rats from both CMS groups (SAL-CMS: 0.56 ± 0.20 CPT; MAM-CMS: 0.55 ± 0.17 CPT) exhibited lower numbers of active DA neurons per electrode track compared with controls (SAL-CON: 1.1 ± 0.19 CPT; MAM-CON: 0.91 ± 0.27 CPT). At baseline, population activity was reduced in the medial VTA tracks of MAM-CMS rats compared with MAM-CON rats (*p* < 0.01; Fig. 4d). Post-FST and AIH, population activity was reduced in the central tracks of MAM-CMS rats compared with MAM-CON rats (*p* < 0.05; Fig. 4e). Post-FST only, population activity was reduced in the central tracks of SAL-CMS rats compared with SAL-CON rats (*p* < 0.05; Fig. 4f).

At baseline (i.e. no FST), there were no effects of MAM (two-way ANOVA; F_1,162_ = 1.031; *p* = 0.31) or CMS (two-way ANOVA; F_1,162_ = 0.173; *p* = 0.68) on firing rate (SAL-CON: 3.91 ± 2.17 Hz; MAM-CON: 3.93 ± 1.99 Hz; SAL-CMS: 3.70 ± 2.84 Hz; MAM-CMS: 4.46 ± 2.38 Hz; Fig. 4g). Post-FST and AIH, no effects of MAM (two-way ANOVA; F_1,157_ = 0.13; *p* = 0.71) or CMS (two-way ANOVA; F_1,157_ = 0.63; *p* = 0.43) were found for firing rate (SAL-CON: 3.71 ± 1.79 Hz; MAM-CON: 4.47 ± 1.85 Hz; SAL-CMS: 4.84 ± 2.30 Hz; MAM-CMS: 3.85 ± 1.93 Hz; Fig. 4h). Post-FST only, no effects of MAM (two-way ANOVA; F_1,218_ = 2.24; *p* = 0.14) or CMS (two-way ANOVA; F_1,218_ = 0.30; *p* = 0.58) were found for firing rate (SAL-CON: 4.03 ± 1.97 Hz; MAM-CON: 3.64 ± 2.37 Hz; SAL-CMS: 4.23 ± 2.01 Hz; MAM-CMS: 3.75 ± 1.67 Hz; Fig. 4i).

At baseline (i.e. no FST), there were no effects of MAM (two-way ANOVA; F_1,162_ = 0.80; *p* = 0.37) or CMS (two-way ANOVA; F_1,162_ = 1.71; *p* = 0.19) on percentage of spikes firing in bursts (SAL-CON: 26.66 ± 27.63%; MAM-CON: 19.96 ± 23.58%; SAL-CMS: 29.98 ± 31.11%; MAM-CMS: 28.54 ± 28.67%; Fig. 4j). Post-FST and AIH, no effects of MAM (two-way ANOVA; F_1,157_ = 0.24; *p* = 0.62) or CMS (two-way ANOVA; F_1,157_ = 0.0043; *p* = 0.95) were found for percentage of spikes firing in bursts (SAL-CON: 29.33 ± 24.28%; MAM-CON: 34.33 ± 25.96%; SAL-CMS: 32.01 ± 28.38%; MAM-CMS: 31.08 ± 24.86%; Fig. 4k). Post-FST only, an effect was found for MAM (two-way ANOVA; F_1,217_ = 5.93; *p* = 0.02) but not CMS (two-way ANOVA; F_1,217_ = 1.06; *p* = 0.30) for percentage of spikes firing in bursts (SAL-CON: 26.43 ± 24.63%; MAM-CON: 21.89 ± 24.61%; SAL-CMS: 33.77 ± 25.99%; MAM-CMS: 21.61 ± 19.00%; Fig. 4l).

### Apomorphine administration reverses stress-induced reductions in VTA DA activity in MAM rats

**Fig. 5:**
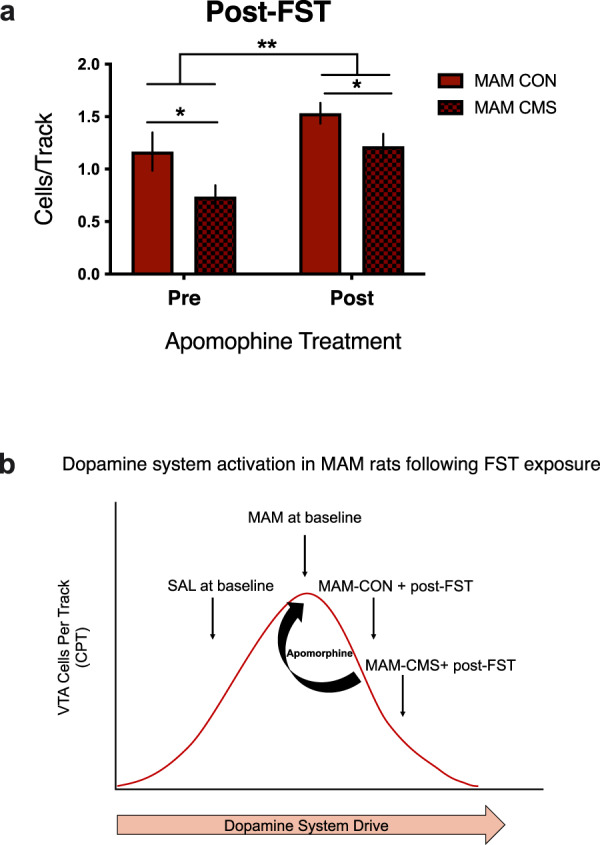
Apomorphine reverses putative FST-induced depolarization block of DA neurons in MAM rats. **a** Post-FST, MAM-CMS rats exhibited blunted VTA population activity compared to MAM-CON rats (main effect CMS: *p* < 0.5, *n* = 4–7per group). In both groups of MAM rats (MAM-CON, MAM-CMS), the majority of FST-exposed animals (67–75%) demonstrated an increase in active cells per track following apomorphine (main effect: pre/post-apomorphine: *p* < 0.01), which is characteristic of reversal of depolarization block. **b** The pattern of DA system activation in MAM rats following stress (FST, CMS) exposure (e.g. blunted response to AMPH, reduced VTA population activity) suggests the possible presence of stress-induced depolarization block, which is reversed by apomorphine. Error bars represent mean ± SEM. Red bars represent MAM animals; checkered bars represent CMS group. *p < 0.05, **p < 0.01.

Depolarization block is a condition whereby over-activation of the VTA results in a reduction in the number of spontaneously active DA neurons (i.e. cells/track-CPT) encountered (attenuated population activity)^38–41^. This effect can be reversed by administration of autoreceptor-selective doses of apomorphine, a DA agonist^38,42–45^. For example, acute stress exposure (i.e. shock) reduces VTA DA neuron activity in MAM rats, and these animals do not exhibit the VTA hyperactivity commonly observed in adult MAM rats due to induction of depolarization block in a proportion of DA neurons, which is reversed by apomorphine administration^42^. To test whether the reduction in DA neuron population activity may have been due to the presence of stress-induced depolarization block, we used a different group of MAM animals (MAM-CON, MAM-CMS) that were exposed to the FST. The changes in the number of VTA DA cells/track following apomorphine treatment varied by condition post-FST exposure (RM two-way ANOVA: main effect of CMS: F_1, 9_ = 7.19; *p* < 0.05; main effect pre vs post-apomorphine: F_1, 9_ = 7.19; *p* < 0.01; Fig. 5a). Post-FST, MAM-CMS animals exhibited reduced number of active DA cells compared to MAM-CON animals. In both groups of stressed (i.e. post-FST) MAM animals (MAM-CON, MAM-CMS), apomorphine administration resulted in an increase in the number of active DA cells/track observed between the right and left hemispheres, which is consistent with a model in which apomorphine administration reversed DA neuron depolarization block (Fig. 5b).

## Discussion

We examined the impact of adult stress exposure, as modeled by CMS, on schizophrenia- and depression-related phenotypes as well as VTA DA neuron activity in MAM rats. We show that MAM rats exhibit CMS-induced increases in FST immobility behaviour (i.e. passive coping) and stress-induced (FST, CMS) reductions in AIH (i.e. blunted DA responsivity). These behavioural alterations were associated with a stress-induced attenuation in VTA population activity. Apomorphine administration restored VTA population activity (# of active VTA DA cells per track) in MAM rats. This is consistent with a model in which apomorphine reversed DA hyperexcitation-induced depolarization block in MAM rats following stress (shock) exposure^42^.

### Effects of prenatal MAM and adult CMS on social behaviour and VTA activity

Social withdrawal is a common negative symptom of schizophrenia that is present across multiple animal models^46–49^. In accordance, we found reduced social motivation, as indexed by less time spent sniffing a cage containing a social stimulus animal, in adult MAM rats compared to adult SAL rats. This finding suggests increased social avoidance^50^ and is consistent with prior reports indicating social withdrawal in MAM rats when tested within an unrestricted (open arena) social interaction test^37,51–53^. Specifically, MAM rats exhibited decreases in active social interaction, defined as the time spent by the two rats in close proximity (≤20 cm) to each other, compared to SAL rats^37,51^. Here we show that MAM rats also exhibit reduced social approach/motivation, as indexed by reduced time spent sniffing the social cage, which is consistent with a recent study showing reduced social interaction behaviours (e.g. sniffing, following, grooming) in MAM rats^52^. Moreover, the social behaviour findings in MAM rats are in agreement with those obtained using other rodent models of schizophrenia, including the neonatal ventral hippocampal lesion, isolation rearing, MK-801 administration and neonatal nitric oxide synthase inhibition models^54–58^.

We found no impact of adult CMS exposure on social motivation in the SAT, which is consistent with a prior study^59^. SAL-CON and SAL-CMS animals exhibited comparable social sniff times. Moreover, SAL-CMS and MAM-CMS rats did not differ from each other. However, MAM-CMS rats exhibited increased social sniff times compared with MAM-CON rats, which may suggest differential effects of CMS on social approach/motivation in MAM vs SAL rats, given that SAL-CMS and SAL-CON exhibited comparable social sniff time. Since MAM-CMS rats exhibited increased social sniff time compared to MAM-CON rats, this finding may suggest a role for hypocortisolism in the social deficits observed in MAM-CON rats, which is consistent with a prior study indicating hypocortisolism in MAM rats^60^. Interestingly, a similar phenomenon has been reported in rats exposed to early life stress, which exhibit a reduction in social behaviour in the SAT that is reversed via administration of the stress hormone corticosterone prior to testing^61^.

We have previously reported DA hyper-responsivity (i.e. increased VTA DA neuron activity) in the MAM model^27,36,43,62–64^. Here, we also found increased numbers of active VTA DA neurons (i.e. population activity) in MAM-CON rats compared to SAL-CON rats. In addition, we show that CMS exposure is sufficient to downregulate DA system activity in MAM rats, although MAM-CMS rats exhibited higher numbers of active VTA DA neurons compared with SAL-CMS rats. Thus, CMS exposure attenuated VTA population activity in both SAL and MAM rats. These results recapitulate prior findings showing CMS-induced decreases in VTA population activity^22,24–26^, which are proposed to reflect reduced DA responsivity^18,21^. We extended these findings to show CMS-induced decreases in DA function in MAM rats.

### Effects of acute and chronic stress on immobility, locomotor response to amphetamine and VTA activity

Passive coping, as indexed by increased immobility or failure to escape, in response to inescapable stress (e.g. forced swim, tail suspension, escapable shock) is commonly reported in animal models relevant to depression^20,21,65^. Although control animals (SAL-CON, MAM-CON) exhibited comparable FST coping responses, adult CMS exposure increased FST immobility and reduced latency to immobility in both SAL and MAM rats. These results are consistent with our previously published work showing that CMS exposure increases FST immobility duration^22,24–26^. Here we extend these findings by showing that FST coping responses in MAM rats are sensitive to the effects of CMS, as MAM-CMS rats exhibited increased passive coping (i.e. immobility) in the FST compared to MAM-CON rats.

Surprisingly, we found reduced locomotor responses to amphetamine in MAM rats. MAM rats that underwent behavioural testing (AIH and FST, counterbalanced order) displayed attenuated locomotor responses to amphetamine compared to SAL animals that also underwent these same tests. Indeed, both MAM-CON and MAM-CMS rats exhibited reductions in the total distance traveled following an amphetamine injection compared to their SAL counterparts; and these alterations were evident within 15–30 min post-injection. Given that MAM rats typically exhibit an increased locomotor response to amphetamine^30,36,63,64,66^, which is thought to reflect augmented DA responsivity; this finding suggests that the acute inescapable stressor employed in this study (FST) was sufficient to induce a persistent change in the level of DA-dependent behavioural activation (i.e. responsivity to amphetamine) in these rats.

Both types of stress exposure (FST, CMS) blocked the DA hyper-responsivity phenotype (i.e. increased VTA population activity) typically seen in stress naïve MAM rats (no FST) or MAM rats that underwent social behaviour testing. Post-FST, MAM-CON rats had comparable numbers of spontaneously active DA neurons compared with SAL-CON animals, suggesting that FST exposure was sufficient to downregulate DA system activity in MAM rats but not in SAL rats. This was also observed in CMS-exposed rats post-FST, in which both groups (SAL-CMS, MAM-CMS) exhibited attenuated population activity compared to standard-housed groups that were tested in the FST (SAL-CON, MAM-CON). No effects of prenatal treatment (SAL vs MAM) or adult housing (CON vs CMS) were found for other parameters of DA system activity (e.g. firing rate, burst firing), suggesting a selective stress effect on VTA DA neuron population activity, which is consistent with our work in two animal models relevant to depression^22,24,25,67^.

### Acute stressors are sufficient to reverse DA hyperresponsivity in MAM rats

Importantly, our results are similar to a prior study showing that MAM rats exhibit enhanced stress-induced DA downregulation (attenuated VTA population activity) following footshock^42^. In that study, MAM rats exhibited persistent alterations in DA system activity following fear conditioning, and fear-induced activation of the DA system was proposed to combine with baseline DA hyperactivity to initiate hyperactivation-induced depolarization block, which will prevent any further activation in response to external stimuli. Indeed, various stressors including footshock can have an activating effect on the DA system, which leads to later downregulation within the same system^18,68^. This was confirmed by subsequent experiments in which the majority of fear-conditioned MAM rats who were administered low-dose apomorphine exhibited increases in VTA population activity, which is consistent with the removal of depolarization block^42^. When placing our findings into this context, a naïve or nonstressed MAM rat typically demonstrates VTA hyperactivity. However, following stress exposure (e.g. shock, FST, CMS) we propose that there is an overdrive of the DA system such that there is a reduction in spontaneous activity and attenuated responsivity. We propose these alterations would manifest as a loss of adequate modulation of DA activity in response to behaviourally salient events (i.e. amphetamine injection), which is supported by our findings showing blunted locomotor responses to amphetamine in MAM animals post-stress exposure (FST, CMS).

In sum, our results show that acute (FST) and prolonged (CMS) stressors reverse DA system hyper-responsivity in MAM rats, which is in agreement with a prior study showing abnormal corticosterone regulation in these rats^60^. Collectively, these data indicate that MAM animals exhibit enhanced stress-induced behavioural and DA system downregulation. These effects were seen across multiple levels (behavioural, electrophysiological) and linked to blunted mesolimbic DA system function. Moreover, these data show that the extent of DA downregulation in MAM rats depends on both the nature and duration of the stressors but can be restored via apomorphine administration, suggesting that stressor-induced attenuation of DA activity is likely mediated via depolarization block.

## Methods

### Animals

Timed pregnant female Sprague-Dawley rats (Envigo, Indianapolis, IN) were obtained at GD15 and housed individually with ad libitum access to food and water. On GD 17, dams received an injection of MAM (diluted in saline, 20 mg/kg i.p.) or vehicle (saline-SAL, 1 ml/kg i.p.). This procedure (i.e. prenatal MAM) induces neurodevelopmental deficits in the cortex and hippocampus of the offspring that resemble those observed in schizophrenia patients^33,69^. The day of birth was considered postnatal day (PD) 0 and litters were weaned on PD 23. Post-weaning, male animals were housed in pairs in a temperature and humidity-controlled facility on a 12 h light/dark cycle (lights on at 7:00 AM; off at 7:00 PM) with food and water available ad libitum until adulthood (>PN 70). Experiments were conducted in males only given our prior results showing that females exhibit enhanced stress sensitivity in response to one of the behavioural tests used (i.e. FST)^25,70^ and that the impact of prepubertal stress is different in males and females^71^. All experiments were performed in accordance with the guidelines outlined in the National Institutes of Health Guide for Care and Use of Laboratory Animals and were approved by the Institutional Animal Care and Use Committee of the University of Pittsburgh.

### Chronic mild stress (CMS)

SAL- and MAM-treated male rats were assigned to standard housing or 4–5 weeks of CMS starting around PD 70–90. The CMS regimen was adapted from our previously published work and consisted of a 4–5-week regimen in which rats were single-housed and randomly presented with 3–4 stressors per week^22,24,25^. Stressors included: food deprivation, water deprivation followed by 1-hour empty bottle presentation, light cycle reversal and/or disruption, cage tilts (45°), overnight stroboscopic lighting (ADJ 58I LED II), damp bedding (200–300 mL of lukewarm water in cage), foreign intruder, white noise (88 dB; continuous) and predator odor exposure (20 ul fox urine for 1 h). Age-matched controls were housed in pairs over the equivalent period of time. All animals underwent weekly cage changes done by the experimenter.

### Behavioural testing

Behavioural tests were selected due to their wide use in preclinical depression and schizophrenia research^10^. After 4–5 weeks of CMS exposure, rats were tested in the SAT. A separate cohort of rats underwent 4–5 weeks of CMS and testing in the FST and AIH in a counterbalanced order. Animals receiving both the FST and AIH received the first test within a week after CMS, and the second test ~7 days later. As an additional control, a separate group of rats from FST only cohorts were tested up to 2 weeks post-CMS. Consistent with our previous work, the FST was administered during the light cycle, whereas the SAT and AIH occurred during the dark cycle^25,71,72^. The experimenter was blinded to the SAL/MAM treatment condition.

### Social approach test (SAT)

Social approach behaviour, as indexed by time spent sniffing a cage containing a novel, younger same-sex conspecific, was assessed in a three-chambered apparatus made from opaque black Plexiglas, in accordance with our prior studies^71,72^. Rats were placed in a smaller center chamber adjacent to two other chambers, each containing a wire cage that allows the test rat to see and smell its content but prevents aggression/sexual behaviours. After a 5 min habituation period to the apparatus, a novel, younger, same-sex rat that had previously been habituated to the wire cage (1 × 15 min) was enclosed inside it and placed in a side chamber. An inanimate object (i.e. toy rat) was placed inside the other wire cage as a novel object control. The test rat was then allowed to explore the entire apparatus and time spent sniffing the receptacle containing the social stimulus (social sniff time) and total number of chamber crossings were recorded for 10 min. Social sniff time was used as a measure of social motivation, in which decreases in social sniff time were interpreted as social avoidance^50^.

### Forced swim test (FST)

The FST took place in a clear Plexiglas cylinder (50 cm high, 20 cm in diameter) filled with water (25 ± 1 °C) up to 38–40 cm, in accordance with our previously published work^25,73^. The FST consisted of two sessions: a 15 min pre-exposure (Day 1—habituation) on the day before the test to ensure stable, high levels of immobility in a 5 min session on the next day (Day 2—test day). Immobility behaviour, defined as making only minor necessary movements to maintain head above water^74^, and latency to immobility were recorded as an indices of behavioural despair/passive coping relevant to depressive-like symptomatology. Water was changed between animals and rats were removed and dried off before being placed back in their home cage on both days.

### Amphetamine-induced locomotion (AIH)

Rats were tested in an open-field chamber in which locomotor activity was determined by beam breaks and recorded with TruScan software (Coulbourn Instruments). Spontaneous locomotor activity was recorded for 30 min. Rats were then injected with D-amphetamine sulphate (0.5 mg/kg, i.p.; Sigma–Aldrich) and locomotor activity was recorded for another 60 min. All animals were tested shortly after the onset of their natural dark cycle (between 7 and 11:00 PM).

### In vivo electrophysiology

#### Surgery and sampling

Single-unit extracellular recordings were performed using an acute preparation in anesthetized rats in accordance with our prior work^25,72^. Rats were anesthetized with chloral hydrate (400 mg/kg, i.p.) and mounted on a stereotaxic frame (Kopf, Tujunga, CA). Body temperature was maintained at 37 °C using a thermostatically controlled feedback heating pad (Fintronics). A burr hole was drilled in the skull overlying the right VTA. Single-barrel glass recording microelectrodes were constructed from 2.0 mm glass tubing by using a Narishige P-5 vertical electrode puller and breaking the tip under microscopic control. Electrodes were filled with 2 M NaCl containing 2% Pontamine Sky Blue dye. Stereotaxic coordinates used for the VTA were 5.3 mm posterior from bregma, 0.6 mm lateral to the midline and 6.5–9.0 mm ventral from the brain surface. The VTA was sampled using a predetermined pattern consisting of 6–9 vertical tracks, each separated by 200 mm, across the antero-posterior (A/P) and medio-lateral (M/L) extent. DA neurons were identified according to well-established electrophysiological features including: location, action potential duration (>2.2 ms), slow firing rate (1–10 Hz), as well irregular and burst firing patterns, with the start of a burst defined as an inter-spike interval ≤80 ms, and the end of a burst defined as a subsequent inter-spike interval >160 ms^75–77^. The activity of each identified DA neuron was recorded for 1–3 min when signal-to-noise ratio exceeded 3:1. Single-unit activity was filtered using a high pass filter at 30 Hz. Three parameters of DA neuron activity were analyzed: (1) the number of spontaneously active DA neurons per electrode track (i.e. population activity), (2) average basal firing rate and (3) the percentage of spikes occurring in bursts.

#### Apomorphine administration

To test for stress-induced depolarization block in MAM animals, a separate cohort of MAM-CON and MAM-CMS animals were exposed to the FST. Post-FST, the VTA was sampled in both right and left hemispheres pre- and post-apomorphine administration (Sigma; 20 μg/kg, i.p.), respectively. Following VTA DA neuron recordings from the right hemisphere (6 tracks), the contralateral VTA was sampled (another 6 tracks) after administration of a low dose of apomorphine. Effective doses of apomorphine were determined by first encountering a spontaneously active DA neuron and then incrementally administering apomorphine (5 μg/kg/increment) until there was an observable change (i.e. approximately a 50% decrease) in baseline firing rate or bursting activity, as previously described by our group^42,43^. Since D2 autoreceptors are more responsive to DA than the post-synaptic receptors, low doses of apomorphine can preferentially stimulate D2 autoreceptors and inhibit dopamine neuron firing^44,45,78–80^. The doses of apomorphine used in this study are consistent with autoreceptor selectivity and have been used previously by our group to test for depolarization block^42,43^.

#### Placement verification

Following the end of the recording session, electrode placements were marked via electrophoretic injection of Pontamine Sky Blue dye from the tip of the electrode (constant negative current, 20–30 min). Rats were euthanized by an overdose of chloral hydrate following marking of electrode placement and brains were removed, fixed for at least 48 hours in 8% paraformaldehyde, cryoprotected in 25% sucrose, and sectioned for histological confirmation of recording sites. Only animals with a minimum of 6 tracks within 0.4 mm of target coordinates were included.

### Statistical analysis

Behavioural results were analyzed using two-way analysis of variance (ANOVA) with prenatal treatment (MAM vs SAL) and adult condition (CON vs CMS) as factors. Post-hoc comparisons (Tukey’s) were performed for ANOVAs showing a significant (*p* < 0.05) interaction and were considered significant when *p* < 0.05. Single-unit neuron activity was analyzed with Powerlab (AD Instruments, Colorado Springs, CO) and Neuroex (NEX Technologies, NexTech Systems, Tampa, FL) software. Electrophysiological results were analyzed using two-way ANOVA with infant treatment, adult condition as factors. Statistical outliers were identified using QuickCalcs Grubbs test (GraphPad) and excluded from analysis. All data supporting the findings of the study are available within the article.

### Reporting summary

Further information on research design is available in the Nature Research Reporting Summary linked to this article.

## Supplementary information

Reporting Summary

## Data Availability

The authors declare that all data supporting the findings of this study are available within the paper and its supplementary information files.
