# Supplementary material for: Adult stress exposure blunts dopamine system hyperresponsivity in a neurodevelopmental rodent model of schizophrenia
Source: Schizophrenia (Heidelb). 2022 Mar 25;8(1):30. doi: 10.1038/s41537-022-00235-x (PMC8956652; doi:10.1038/s41537-022-00235-x)
Supplement: Supplementary file 1 — Reporting Summary [file 41537_2022_235_MOESM1_ESM.pdf]

nature portfolio

Corresponding author(s): Millie Rincón-Cortés

Last updated by author(s): 1-14-2022

Reporting Summary

Nature Portfolio wishes to improve the reproducibility of the work that we publish. This form provides structure for consistency and transparency in reporting. For further information on Nature Portfolio policies, see our Editorial Policies and the Editorial Policy Checklist.

Statistics

For all statistical analyses, confirm that the following items are present in the figure legend, table legend, main text, or Methods section.

n/a	Confirmed	
☐	☒	The exact sample size (*n*) for each experimental group/condition, given as a discrete number and unit of measurement
☐	☒	A statement on whether measurements were taken from distinct samples or whether the same sample was measured repeatedly
☐	☒	The statistical test(s) used AND whether they are one- or two-sided<br>*Only common tests should be described solely by name; describe more complex techniques in the Methods section.*
☒	☐	A description of all covariates tested
☒	☐	A description of any assumptions or corrections, such as tests of normality and adjustment for multiple comparisons
☐	☒	A full description of the statistical parameters including central tendency (e.g. means) or other basic estimates (e.g. regression coefficient) AND variation (e.g. standard deviation) or associated estimates of uncertainty (e.g. confidence intervals)
☐	☒	For null hypothesis testing, the test statistic (e.g. *F*, *t*, *r*) with confidence intervals, effect sizes, degrees of freedom and *P* value noted<br>*Give P values as exact values whenever suitable.*
☒	☐	For Bayesian analysis, information on the choice of priors and Markov chain Monte Carlo settings
☒	☐	For hierarchical and complex designs, identification of the appropriate level for tests and full reporting of outcomes
☒	☐	Estimates of effect sizes (e.g. Cohen's *d*, Pearson's *r*), indicating how they were calculated

Our web collection on statistics for biologists contains articles on many of the points above.

Software and code

Policy information about availability of computer code

Data collection	PowerLab 6/30 LabChart
Data analysis	Single-unit neuron activity was analyzed with Powerlab LabChart v8(AD Instruments, Colorado Springs, CO) and Neuroex (NEX Technologies, NexTech Systems, Tampa, FL) software. Statistical analyses were conducted using GraphPad Prism v9.0.

For manuscripts utilizing custom algorithms or software that are central to the research but not yet described in published literature, software must be made available to editors and reviewers. We strongly encourage code deposition in a community repository (e.g. GitHub). See the Nature Portfolio guidelines for submitting code & software for further information.

Data

Policy information about availability of data

All manuscripts must include a data availability statement. This statement should provide the following information, where applicable:

- Accession codes, unique identifiers, or web links for publicly available datasets
- A description of any restrictions on data availability
- For clinical datasets or third party data, please ensure that the statement adheres to our policy

All data supporting the findings of the study are available within the article.

Field-specific reporting

Please select the one below that is the best fit for your research. If you are not sure, read the appropriate sections before making your selection.

☒ Life sciences ☐ Behavioural & social sciences ☐ Ecological, evolutionary & environmental sciences

For a reference copy of the document with all sections, see nature.com/documents/nr-reporting-summary-flat.pdf

Life sciences study design

All studies must disclose on these points even when the disclosure is negative.

Sample size	Sample sizes were selected based on our previously published work on stress effects in MAM rats and depolarization block studies (Sonnenschein et al. 2019, Gill et al. 2018, Valenti et al. 2011).
Data exclusions	Ellectrode placements were verifie through histology and only animals with a minimum of 6 tracks within 0.4mm of target coordinates were included in the study (see Methods). Statistical outliers were identified using QuickCalcs Grubbs test (GraphPad) and excluded from analysis.
Replication	The VTA findings of Fig. 2 are replicated in Fig. 4. We also added an experiment (per reviewer request) that replicates our prior cohort findings regarding FST and CMS effects of VTA population activity in MAM rats.
Randomization	Allocation to experimental groups was randomized.
Blinding	The experimenter was blind to with regard to the SAL/MAM treatment condition during behavioral testing and electrophysiological recording data collection. CMS animals are housed in a different room than all other rats so blinding during the collection of this data is nearly impossible.

Reporting for specific materials, systems and methods

We require information from authors about some types of materials, experimental systems and methods used in many studies. Here, indicate whether each material, system or method listed is relevant to your study. If you are not sure if a list item applies to your research, read the appropriate section before selecting a response.

Materials & experimental systems

n/a	Involved in the study	
☒	☐	Antibodies
☒	☐	Eukaryotic cell lines
☒	☐	Palaeontology and archaeology
☐	☒	Animals and other organisms
☒	☐	Human research participants
☒	☐	Clinical data
☒	☐	Dual use research of concern

Methods

n/a	Involved in the study	
☒	☐	ChIP-seq
☒	☐	Flow cytometry
☒	☐	MRI-based neuroimaging

Animals and other organisms

Policy information about studies involving animals; ARRIVE guidelines recommended for reporting animal research

Laboratory animals	Rat, Sprague Dawley, Males, Adult
Wild animals	The study did not involve wild animals.
Field-collected samples	The study did not involve field-collected samples.
Ethics oversight	All experiments were performed in accordance with the guidelines outlined in the National Institutes of Health Guide for Care and Use of Laboratory Animals and were approved by the Institutional Animal Care and Use Committee of the University of Pittsburgh.

Note that full information on the approval of the study protocol must also be provided in the manuscript.
